# American Medical Association—Plans for Its Improvement

**Published:** 1872-12-01

**Authors:** 


### MEDICAL EXAMINER.

*A Semi-Monthly Journal of Medical Sciences.*

EDITED BY

N. S. DAVIS, M. D., AND F. H. DAVIS, M. D.

Chicago, December 1st, 1872.

### EDITORIAL.

### AMERICAN MEDICAL ASSOCIATION—PLANS FOR ITS IMPROVEMENT.

The leading editorial in the Boston *Medical and Surgical Journal*, for July 25th, 1872, contains the following suggestions in regard to the plan of organization of the "American Medical Association :"

"Hitherto, the Association has shown, as might have been anticipated, that men, as a general thing, do not value, but rather avoid, as conferring no honor or satisfaction even, positions which, as we have intimated, any one, however inexperienced, incompetent or unfit, can have for the asking. Herein is its present weakness, which must be removed before any hope of improvement can be entertained. In future, then, let the Association have or rather consist of a working Board, a true representative body, of limited numbers, membership in which shall be the reward of professional ability, usefulness and eminence. Two from each State would be a sufficiently large number for all practical purposes. Let this Board be called the National Council of the American Medical Association. Let this Council meet once a year at some convenient place, say Washington, or as the Board might from time to time determine, and continue in session sufficiently long to attend to all legitimate business, scientific and professional, everything in fact in the interests and needs of the profession throughout the Union—giving such advice, recommending such rules, issuing such "Transactions," as would be for the elevation and advancement of the whole profession. Let the term of service for these two National Councillors be six years — one to be chosen every three years; and to place them as much as possible above temporary influences and purely local interests, let them be chosen by a State Council, which for this and other duties, may be constituted and elected as follows :

Let each of the several State Societies choose from its most experienced and best qualified men in number equal, say to that of the State's Representatives in Congress ( one-third each year ), and, as far as convenient, to obtain representation of all parts of the State—one from each Congressional District. Let these hold office for three years, and form what may be called the Local or State Council of the American Medical Association. They could meet yearly, or oftener if found useful, to consider, primarily, professional matters which in the State are, or may be, of National concernment, and to make selections of such as should be carried before the National Board — in short, to do all the work of a State Committee. Each of these State Boards should, once in three years, choose one of the National Councillors, as before mentioned, from their own Board or at large, wherever the best qualified could be found. As in these State Boards the chief preparative work should be done, while its revision and completion should be left to the sober judgment and more deliberate action of the National Board, the National Councillors might have seats in the State Board — without vote — in order that they might know all the arguments and circumstances relating to any case, and the wishes of the State Board, in full, to guide their own action in the National Board.

Should the plan work successfully, as we have no doubt it would, eventually the State Societies might, if then thought advisable, without changing name or management in any other respect, become branches, and their Districts sub-branches of the National organization, retaining all the desirable features now possessed; and thus give to each of their members admitted by nomination of county societies ( or District societies ) after two or more year's practice and probation ( if thought best ), an additional bond of union with the whole profession in the country, and an additional *title* to the respect of the community at large.

In such event the, National Council could arrange for general meetings ( every year or second year, and in different parts of the country ) for all members who might choose to attend. At these meetings there might be orations in medicine, in surgery, and in other departments, reports, communications, discussions, and consideration of other matters of professional interest ; while all the evils of the present organization would be avoided, by the exclusion of all matters of busines or control, which would continue in the hands of the National Council, and not be agitated at any of these general meetings."

It appears that the leading ideas embraced in the foregoing article, are, that the Association, as at present organized, is not sufficiently representative; that membership is so easily obtained as to make it regarded of little value; that the attendance of the same members is too short or irregular to give them the necessary experience, or to impart to the proceedings of the Association the desired stability and uniformity; and that the annual meetings are too large, and their sessions too brief, to permit efficiency in the transaction of important business. To remedy these defects, it is proposed to create a National Council, composed of two delegates from each State, which would sufficiently limit the number, and make the body strictly representative. To secure permanency, each delegate is to hold office six years, allowing one to be elected from each State every three years. It is suggested that the delegates from each State be appointed by a State Council, composed of one delegate from each congressional district, the latter to be appointed by the State Medical Societies. These State and National Medical Councils, are to have the exclusive control of purely business matters, including all questions of education, literature, ethics, legislation, and publication of transactions, leaving the general meetings of the State and National Associations to be devoted wholly to scientific and social purposes. The leading idea underlying these suggestions, that we ought to have a smaller, more stable, and more truly representative body, to take charge of all strictly business matters, is not new, but has been suggested by several individuals, and at different periods of time. Dr. Isaac Hays, of Philadelphia, at the first meeting, when the original plan of organization was under discussion, urged such amendments as would place all the business matters of the Association in a council of limited number, elected in such a manner as to ensure stability, while the membership of the Association might be almost indefinitely increased, and the general meetings devoted exclusively to scientific and social purposes.

At the meeting in San Francisco, 1870, Dr. S. G. Moses, of St. Louis, sent a communication, recommending the engrafting upon the Association the organization of a National Academy of Medicine, consisting of a limited number of representatives, which should elevate and regulate the standard of education, by providing suitable boards of examiners, who should examine all applicants, and give to such as were found qualified, a Degree, that would be independent of and higher than any mere college diploma. The communication was referred to the standing Committtee on Medical Education, and reported on at the meeting in Philadelphia, last year. In their report, the committee, instead of recommending the plan of Dr. Moses, proposed the convening of a Medical Congress, consisting of two representatives from each State, and one from each medical college in the country, all of whom should be appointed by the Association. These various propositions, all aiming at the establishment of a national organization, far less numerous in membership, more stable, and more truly representative, are sufficient to indicate an increasing sentiment in the profession, favoring such an object.

Yet, all the plans thus far proposed, are so defective in details as to render it difficult to judge of their merits. It is very easy to say, with the Boston *Medical and Surgical Journal*, that the present Association is too large and unstable in its membership, and its annual meetings too short to permit efficiency and judiciousness in the transaction of important business. It is equally easy to suggest, that a National Council should be appointed, consisting of two from each State, selected from the wisest and best men in the profession; and that a State Council should exist in each State, consisting of one delegate from each congressional district, and selected with such care as to make each a true representative of the profession in his district. But when we inquire *how* the members of the National and State Councils shall be appointed, in order to secure the actual selection of the *best* men, the answer is not so easily given. Dr. Moses, in proposing the establishment of a National Academy, says nothing about how the members should be elected.

The Committee on Medical Education, at the last annual meeting, in proposing a National Congress, composed of two from each State, suggested their selection by the Nominating Committee, and election by the Association. The Boston *Journal* suggests, that the members of the National Council be appointed by the State Councils, and hold their offices six years, one being chosen every three years, while the members of the State Council should be selected by the State Societies.

Practically, then, all the propositions thus far made, contemplate the selection of delegates to the new organizations, by either the National or State Societies.

But does the experience of the past or the practical working of these societies, justify the conclusion that they would always, or even generally, select the best men? Is there not as good an opportunity for interested, ambitious, and unfit members to electioneer and make combinations to secure their election in these societies, as in any of the other relations of human society? And when appointed, would their acts have any more weight, or command any more ready obedience, than would the same acts if performed by the present societies? These are questions worthy of careful consideration, but which we do not propose to answer at present.

Again, if the National and State Councils, are to consider "all legitimate business, scientific and professional, everything, in fact, in the interests and needs of the profession throughout the Union—giving such advice, recommending such rules, issuing such 'Transactions,' as would be for the elevation and advancement of the whole profession," as stated by our Boston cotemporary, what need is there of retaining our present National Association, or even the State Societies in existence? The election of the delegate to the State Council could as well devolve on the joint action of whatever local societies might exist in each congressional district, and then neither the State Societies nor the National Association would have anything of importance left to occupy their sessions, except social intercourse. It is true, that a part of the time of each meeting might be spent in listening to addresses on different departments of medicine, or papers; but if all action on such papers, and all questions of medical polity must be referred over to the State and National Councils respectively, we doubt whether there would remain sufficient interest to keep these organizations in active existence.

If we understand the report of the Committee on Medical Education correctly, the National Congress therein suggested, was to be only a temporary organization, for a special purpose. That purpose was, to agree on a carefully-devised scheme of medical education for the whole Union, and then enforce its adoption, by thereafter refusing membership in the Association, to all institutions that did not comply.

But is it likely that such a Congress would devise any better scheme of education than the one agreed upon by the Convention of Delegates from medical colleges, which was held in Cincinnati, in 1867, and re-affirmed by another convention of college delegates, in Washington, in 1870; and twice unanimously approved by the Association? If not, would it not be better to turn attention at once to the measures necessary to enforcing this scheme, instead of consuming time in devising another? We do not clearly see how the recommendations of another convention or congress would have any more practical force, or would be more easily executed, than those of the conventions already held. Having thus noticed the several propositions which have been made by parties who are dissatisfied with the progress of the past, the questions remain, whether our present State and National organizations are susceptible of important improvements, and whether there is any feasible plan by which a higher standard of medical education can be enforced? These we will endeavor to answer in our next issue.

